# Meeting preschool screen time recommendations: which parental strategies matter?

**DOI:** 10.3389/fpsyg.2023.1287396

**Published:** 2023-11-06

**Authors:** Caroline Fitzpatrick, Emma Cristini, Jonathan Y. Bernard, Gabrielle Garon-Carrier

**Affiliations:** ^1^Department of Preschool and Elementary School Education, Université de Sherbrooke, Sherbrooke, QC, Canada; ^2^Department of Childhood Education, University of Johannesburg, Johannesburg, South Africa; ^3^Centre for Research in Epidemiology and Statistics (CRESS), Université Paris Cité and Université Sorbonne Paris Nord, Inserm, INRAE, Paris, France; ^4^Department of Psychoeducation, Université de Sherbrooke, Sherbrooke, QC, Canada

**Keywords:** screen time, screen use, guidelines, parent mediation, parental monitoring, preschooler, early childhood

## Abstract

**Background:**

High levels of screen use by preschoolers may contribute to adverse health and developmental outcomes. Little is known about which parental strategies may be protective against intensive screen use by children. Our aim is to estimate whether parent strategies for mediating child screen time including restrictive and instructive mediation and social coviewing, predict preschooler adherence to the screen time recommendation of ≤1 h/day during the COVID-19 pandemic. We also examine if parent restrictive mediation interacts with child temperament characteristics.

**Methods:**

Our sample is composed of 315 Canadian parents of preschoolers surveyed during the COVID-19 pandemic. Parents provided measures of child adherence to screen time guidelines at ages 3.5 (baseline) and 4.5 years. Parents also reported how often they used restrictive and instructional mediation, and social coviewing to manage their child’s screen use. Control variables include child sex and temperament (effortful control, negative affectivity, extraversion), educational attainment, and parenting stress at age 3.5.

**Results:**

A logistic regression revealed that parental restrictiveness was associated with a 4 time increase in the odds of adherence to screen time guidelines, OR = 4.07 (95% CI [1.70–13.03]). Parental social coviewing decreased the chances of adherence by 80% (OR = 0.20, 95% CI [0.09–0.48]). Furthermore, children not meeting recommendation at 3.5 were 98% less likely to respect the recommendation 1 year later (OR = 0.02, 95% CI [0.01–0.07]). Results were adjusted for child sex, temperament, baseline screen time, and parent education and stress The interaction between the restriction mediation and child temperament on later screen time was not significant.

**Conclusion:**

Our results indicate that some parental strategies may be more effective than others for managing preschooler screen time. Parent use of restrictive mediation was most likely to forecast child adherence to later screen time recommendations. The present results may contribute to the development of targeted family-based interventions designed to promote healthy development from a young age.

## Introduction

The amount of time preschoolers accumulate in front of screens can undermine later fitness, sleep quality, and school readiness (Jones et al., 2013; Madigan et al., 2019; Lan et al., 2020). Since habits and behaviors developed in early childhood are likely to be carried forward in later life stages, helping children develop healthy screen time habits during the preschool years may promote lifelong health and decreased morbidity (Jones et al., 2013). The World Health Organization currently recommends that children between the ages of 2 and 5 be exposed to a maximum of 1 h of daily screen time (World Health Organization, 2019). Research conducted prior to COVID-19 pandemic has found that half or less of Canadian children respect these guidelines (Carson et al., 2013; Tamana et al., 2019). The increased sedentariness of young children is of particular concern given that the cardiorespiratory health of children between the ages of 6 and 17 has been declining over the last decades (Leone et al., 2023). Furthermore, there has been a dramatic raise in children that are overweight or obese internationally (Ng et al., 2014).

Child screen use occurs within their larger family ecology (Barr, 2019). As such, parental strategies represent promising intervention targets for helping children develop healthy screen use habits. Based on previous research, parents use three strategies to manage child screen use (Valkenburg et al., 1999). A first strategy is the use of *restrictive mediation* which includes parents setting limits on child screen time and establishing rules surrounding what contents children may view. A second strategy, *instructive mediation* refers to parents discussing the content of media with children in an attempt to help foster learning. Finally, *social coviewing* involves parent viewing media with children for enjoyment purposes rather than educational ones (Valkenburg et al., 1999; Livingstone and Helsper, 2008).

Few studies have prospectively examined how parent strategies for managing child screen time contribute to later adherence to screen time recommendations and existing studies have provided mixed results. One study with school-aged children and adolescents has found that parent use of restrictive strategies can help reduce child screen time and prevent excessive internet use (Fu et al., 2020). In contrast, another study has found that restrictive practices may lead adolescents to engage in more screen use (Hefner et al., 2019). According to one qualitative study on 5–6 years-olds, rule setting appears to be a promising approach for managing screen time (Jago et al., 2016). Furthermore, cross-sectional data support a negative association between parental restrictive mediation and preschooler screen time (Fitzpatrick et al., 2022a). Currently, it also unclear if parenting contributes to child media habits, or if parenting strategies are shaped by children’s screen use habits and preferences. That is, parents who perceive their child’s screen use as more problematic may be more likely to adopt restrictive mediation. For this reason, prospective studies that control for baseline child screen time are helpful for shedding light on the direction of these associations.

Active forms of mediation like instruction and social coviewing have been shown to help protect 8–14 years-old children from problematic internet and phone use (Hefner et al., 2019). Furthermore, active mediation by parents has been linked to lower odds of excessive internet use cross-sectionally in adolescents (Kalmus et al., 2015). Finally, according to a meta-analysis, both restrictive mediation and coviewing may reduce child screen time, whereas parental instruction was associated with reduced child aggression and substance use in older children and adolescents (Collier et al., 2016). Research on the benefits of restriction, instruction, and social coviewing on younger children remains sparse.

In addition to child age, individual differences in child self-control may also moderate associations between restrictive practices and child screen time habits. For instance, Lee (2013), found that adolescents with lower levels of self-control may benefit more from restrictive mediation than those with higher levels of self-control. These associations remain unexamined with younger preschool-aged children. In early childhood, the ability to exercise self-control over emotions, behavior, and attention are strongly influenced by children’s temperamental characteristics which include dimensions of effortful control (ex., reflecting attentional focusing and inhibitory control), negative affectivity (ex., frequency of expression of anger and frustration), and extraversion (ex., tendency to act impulsivity) (Putnam and Rothbart, 2006). For this reason, the extent to which restrictive mediation, which involves the implementation of rules and restrictions, may interact with child temperament to influence child screen use habits.

To guide the development and creation of effective interventions to reduce sedentary time among preschoolers, it also remains important to consider parenting strategies in the context of the larger family ecology (Barr, 2019). Some research suggests that boys are exposed to more screens than girls (Rideout et al., 2022). Furthermore, children with more difficult temperaments may also elicit more screen time from caregivers (Coyne et al., 2021). In terms of parent characteristics, educational attainment and distress are likely to play an important role in shaping child screen use (Hartshorne et al., 2020). The recent COVID-19 pandemic provides an opportunity to study parent mediation in the context of increased family distress. In particular, stay at home orders and disruptions to family routines led to a sharp increase in screen use during this time (Hartshorne et al., 2020; Coyne et al., 2021).

The objective of the present study is to examine how parental mediation strategies at the age of 3.5 years predict later child adherence to screen time guidelines at age 4.5 years. We examine these associations while controlling for baseline screen time, sex, and temperament and parent education and parenting stress. Given the results of prior research, we hypothesize that more parental restriction, social coviewing, and instructive mediation will each be associated with lower odds of children exceeding 1 h/day of screen time. Finally, given that previous research has found that child self-control can interact with restrictive mediation, we examine possible heterogeneity in child screen time by examining if parent use of restrictive mediation interacts with child temperamental characteristics.

## Methods

### Study sample

The present study employed a longitudinal correlational design undertaken to better understand the consequences of child screen media use during the pandemic. At the start of our study, children were between the ages of 2 and 5 (*N* = 315, mean age = 3.46). Follow-up took place 1 year later (*N* = 265, mean age = 4.50). Our community-based sample was recruited using convenience sampling methods. More specifically, we recruited families through preschool and pre-kindergarten classes using posters, flyers, and signup sheets, a Facebook page, and newspaper and radio advertisements broadcast across the province of Nova Scotia, Canada. In the vast majority of cases, mothers were the primary respondent (*N* = 295, 93.4%). Our sample contained slightly more boys than girls (54 vs. 46%). Parents in our sample were mostly Canadian born (91%), married (82%), white (90.5%), and English speaking (88.1%). Parents received a 50$ gift card as compensation at each wave of data collection. Parents also provided informed consent to participate at each wave of the study. This project received ethics approval from Université Sainte-Anne and Université de Sherbrooke’s internal review boards.

### Data collection procedure

Parents completed the CAFÉ Assessment of family media exposure online remotely in the Spring of 2020. This assessment has been described in detail in a previous study (Barr et al., 2020). The CAFÉ assessment includes questions on child sex and screen time habits, parental education, as well as questions on parental mediation of child screen time habits. For the purpose of this study, items measuring child temperament were added to our online questionnaire. All study measures are described below.

### Measures: outcomes (age 4.5 y)

Child average daily screen time. Parents completed the Media Assessment Questionnaire (MAQ, Barr et al., 2020) online, to provide estimates of the amount of time their child spent engaged in the following screen-based activities on weekdays and weekend days separately: (1) watching TV or DVDs; (2); using an iPad, tablet, LeapPad, iTouch, or similar mobile device (excluding smartphones); or (3) using a smartphone; (4) playing video games on a console; (5) using a computer. Possible responses include: (1) Never; (2) Less than 30 min; (3) 30 min to 1 h; (4) 1–2 h; (5) 2–3 h; (6) 4–5 h; (7) more than 5 h. All categorical responses were transformed into a variable reflecting the number of hours spent with each type of activity. More specifically we used the mid-point of each response range. For the category “5 or more hours a day” a conservative estimate of 5 h was used. A daily estimate for each screen-based activity was estimated by multiplying weekday estimates by 5, weekend day estimates by 2, and dividing the total by 7. We then calculated an estimate of child daily screen time by summing across all screen-based activities. A similar approach to measuring screen time has been used in previous publications (Fitzpatrick et al., 2022b; Almeida et al., 2023; Fitzpatrick et al., 2023). Finally, scores were dichotomized to distinguish children who followed recommendations (1 = ≥1 h/day) vs. those that did not (0 = <1 h/day). The same strategy was used to measure children’s screen time at age 3.5 years.

### Predictors (age 3.5 y)

Parental mediation practices. Parents reported how frequently they engaged in restrictive and instructive mediation and social coviewing. Items were from Valkenburg et al.’s scale (Valkenburg et al., 1999) which was created and validated to measure parent mediation strategies of child screen use. All items were rated using a Likert scale, with response options ranging from: 1 (never); 2 (rarely); 3 (sometimes); and 4 (often). Items for *restrictive mediation* include: set specific viewing hours for your child; restrict the amount of child viewing; tell your child to turn off the TV when they are watching an unsuitable program; tell your child in advance the programs they may watch; forbid your child to watch certain programs, alpha = 0.64; *instructive mediation* includes: try to help the child understand what s/he sees on TV; point out why some things actors do are bad; explain what something on TV really means; explain the motives of TV characters; point out why some things actors do are good, alpha = 0.86. Finally, *social coviewing* includes: watch together because you both like the program; laugh with the child about the things you see on TV; watch together because of a common interest in a program, alpha = 0.85.

### Covariates (age 3.5 y)

Child characteristics. Parents reported child age, sex (2 = girl, 1 = boy), and temperament. Temperament was assessed using the Children’s Behavior Questionnaire (Putnam and Rothbart, 2006). This instrument captures three distinct child temperament factors: effortful control, negative affectivity, and surgency/extraversion. *Effortful control* includes child inhibitory control and attention focusing (e.g., Can wait before entering into new activities if s/he is asked to). *Negative affectivity* includes measures of child anger/frustration (e.g., Child gets angry when told s/he has to go to bed). *Surgency/extraversion* reflects the child’s level of shyness (reverse coded) and impulsivity (e.g., Child usually rushes into an activity without thinking about it) indicating higher levels of impulsivity and activity. Higher scores indicate higher levels of the temperamental factor. The third factor, effortful control, refers to the child’s abilities to self-regulate their level of reactivity. The short version uses a 7-point Likert scale ranging from 1 (*extremely untrue of your child*) to 7 (*extremely true of your child*). The internal consistency coefficients are 0.79 for Effortful control, and 0.84 and 0.84 for Negative affectivity and Surgency/extraversion, respectively.

Parent characteristics. Responding parents completed the parenting distress subscale of the Parent Stress Index (Abidin, 2012). This measure includes 12 items that capture parent negative feelings toward their relationship with their child (i.e., I find myself giving up more of my life to meet my child’s needs than I ever expected), rated on a 5-point Likert scale. Likert scale response options ranged from: 1 (strongly disagree); 2 (disagree); 3 (not sure); 4 (agree) or 5 (strongly agree). Parent responses were summed to create a total score, Cronbach’s alpha = 0.85. Parents also provided information on their level of educational attainment. Responses were dichotomized as either: (1) High school or college vocational; (2) Undergraduate or Graduate degree.

### Statistical analyses

We begin by conducting preliminary descriptive and bivariate analyses between our main predictor and outcome variables. To address our research objective, we first estimate a multinomial logistic regression between parental mediation strategies (restrictive and instructive mediation, social coviewing) at age 3.5 years and adherence to screen time recommendations at age 4.5 years. Associations are estimated controlling for child sex, screen time, temperament, and parent education and stress. To address heterogeneity in the association between parental restrictive mediation and adherence to screen time recommendations, we add an interaction term between child effortful control and restrictive mediation which is then added to the regression model. All statistical analyses were carried out with SPSS (version 27). Consistent with previous research examining behavioral health and psychological outcomes, the alpha level was set at 0.05.

## Results

### Descriptive statistics and bivariate correlations

Descriptive statistics and frequencies for continuous and categorical variables are presented in Tables 1, 2. In total, 14% (*N* = 44) spent ≤1 h per day with screens at 3.5 years and 20% (*N* = 52) of our sample spent ≤1 h per day with screens at 4.5 years. Chi-square analyses revealed that adherence to guidelines at ages 3.5 and 4.5 (scored dichotomously), were positively related, with adherence at 3.5 forecasting greater adherence one year later *χ*^2^(1, *N* = 265) = 94.13, *p* < 0.0001. Furthermore, Kendall-Tau correlations were performed to estimate bivariate associations between continuous parent strategies and the dichotomous outcome. These analyses revealed that more restrictive mediation was associated with greater adherence at 4.5 (*r* = 0.28, *p* < 0.001) whereas social coviewing negatively associated with later adherence to screen time recommendations (*r* = −0.16, *p* < 0.001). Finally, Pearson’s correlation revealed that parental mediation practices were moderately correlated with each other. Social coviewing was correlated with more instructive mediation (*r* = 0.40, *p* < 0.001) and more restrictive mediation was associated with more instructive mediation (*r* = 0.26, *p* < 0.001). Restrictive mediation was not associated with social coviewing.

**Table 1:** Descriptive statistics for continuous variables.

	Mean (SD)	*N*
Restrictive mediation	3.18 (0.65)	311
Social coviewing	3.07 (0.63)	311
Instructive mediation	3.13 (0.65)	308
Effortful control	4.70 (0.85)	315
Negative affectivity	3.61 (0.90)	315
Extraversion	4.27 (0.98)	315
Parenting stress	18.19 (5.60)	315

**Table 2:** Frequencies for categorical variables.

	% (*N*)
Age 4.5 y	
Screen time	
1 h or less/day	20 (53)
Age 3.5 y	
Screen time	
1 h or less/day	14 (44)
Child sex	
Girls	46 (145)
Parent education	
High school/vocational	26 (81)

### Missing data

In total 84% of our sample had complete data at both time points. Children with parents with a university degree were more likely than those without to remain in our sample at the second wave, *χ* (1)2 = 4.24 *p* = 0.039. Child sex, screen time, temperament, and parenting stress were unrelated to participant attrition. Little’s test provided evidence that our data was met the missing completely at random hypothesis. As such, following best practices for treating missing data, we conducted analyses on the pooled estimates from 5 imputed data sets (Cummings, 2013).

### Predicting adherence to screen time recommendations

As presented in Table 3, the logistic regression model revealed that a 1-point increase on the parental restriction scale at age 3.5 was associated with an increased odds of adhering to screen time recommendations 1 year later (OR [95% CI] = 4.07 [1.70–13.03]). In contrast, parental social coviewing was associated with an 80% decrease in the odds of meeting screen time recommendations (0.20 [0.09, 0.48]) and children exceeding screen time recommendations were 98% less likely to follow recommendations one year later (0.02 [0.01, 0.07]). Finally, children who scored higher on the temperamental dimension of negative affectivity were 51% less likely to spend ≤1 h/day with screens (0.49 [0.27, 0.87]). Instructive mediation did not contribute to child screen time nor did sex, effortful control, extroversion, and parental education and stress.

**Table 3:** Adjusted logistic regression estimating the probability of children meeting screen time recommendations at age 4.5 from parental mediation practices at age 3.5 (imputed data).

	Child screen time (age 4.5) (1 = follows recommendations 0 = Does not follow recommendations)	
	Odds ratio (95% CI)	*P*-value
Child sex		
Girl	0.70 (0.29–0.1.73)	0.440
Boy (reference)	–	–
Child temperament		
Effortful control	0.65 (0.34–1.26)	0.201
Negative affectivity	0.49 (0.27–0.87)	0.015
Extraversion	0.76 (0.47–1.24)	0.273
Screen time (age 3.5)		
More than 1 h/day	0.02 (0.01–0.07)	*p* < 0.0001
1 h or less/day (ref)	–	
Parental mediation		
Coviewing	0.20 (0.09–0.48)	*p* < 0.0001
Restrictive	4.07 (1.70–13.03)	0.003
Instructive	1.82 (0.82–4.02)	0.139
Parental education		
University degree	2.86 (0.75–10.90)	0.123
HS/vocational (ref)	–	
Parenting stress	1.00 (0.92–1.10)	0.935

Male is coded as 1 and female as 2. Education is coded as 0 = High school vocational, 1 = University/graduate.

### Moderation

We considered the extent to which the strength and direction of the observed association between parental restrictive mediation and adherence to screen time recommendations may differ based on child temperamental characteristics. More specifically, we examined whether the interactions of child effortful control, negative affectivity, and extraversion and restrictive mediation at 3.5 years contributed to following screen time recommendation at 4.5 years. In a second regression model, the interaction of child effortful control and parent restrictive mediation was entered as predictor. The interaction term was not significantly associated with odds of adhering to screen time recommendations (all *p*’s > 0.05 results not shown).

## Discussion

Our results indicated a low level of adherence to screen time recommendations among 3 (14%) and 4 years-olds (20%) during the COVID-19 pandemic. These estimates are comparable to other estimates collected from Canadian samples during the pandemic (Madigan et al., 2022). In the present study, we found that parental restrictive mediation, which involves behaviors like establishing specific viewing hours for the child or restricting how long they can view screen media, was associated with a 4 times greater chance of respecting the recommendation of ≤1 h of daily screen time. This association was observed in the context of the pandemic, above and beyond child sex and baseline screen time, temperament, and parental education and stress. Since the pandemic represented a time of increased strain and distress for families, replications are warranted to examine the extent to which parental restrictions as well as instructive mediation and co-viewing may contribute to child screen time under more typical circumstances.

Unlike previous research (Lee, 2013), we did not find evidence of an interaction between child effortful control, extraversion, and negative affectivity on later screen time. This suggest that restrictive approaches may be an effective strategy with young children, regardless of their temperamental characteristics and family context. Although child temperamental characteristics did not modify the association between parental restrictive management and later child screen time, child negative reactivity was associated with more screen time at the age of 3. This finding supports previous studies indicating that children with more challenging behaviors at age 3 are likely to be exposed to more screen time by the age of 5 (Neville et al., 2021).

In the present study, instructive mediation, which involves discussing the contents of screen media to stimulate critical thinking, was not related to child screen time habits. This may be the case because instructive mediation contributes to positive outcomes mainly in older children and adolescents as they become better able to think critically and self-regulate their behaviors (Nathanson, 2002; Fu et al., 2020). Furthermore, discussing the actions and motives of characters may exercise a protective effect when children are viewing traditional television programs but less so when they are using a tablet or mobile device for other types of activities. Social coviewing, which involves parents and children sharing the screen viewing experience without attempts to critically discuss the contents, reduced the chances of following the recommendation. A possible explanation for this finding is that coviewing of media with parents may be perceived as a parental endorsement of screen time, whereas restrictive mediation may be perceived by children as parental disapproval of screen media viewing (Nathanson, 2001).

The preschool years may present a window of opportunity during which restrictive practices may be especially effective. That is, self-control develops rapidly during the preschool years and is likely to benefit from parental scaffolding in the form of preestablished rules and routines. In contrast, the use of restrictive mediation may backfire with older children (Nathanson, 2002). For instance, a study of adolescents whose parents used restrictive mediation of their television viewing reported more positive attitudes towards television content.

Future research could attempt to better understand which factors contribute to parent’s use of restriction and rule setting surrounding their child’s screen use. Previous research has found that parents’ attitudes towards screen media, media literacy, and beliefs surrounding the impact of screen media on their child’s behavior are related to media rule setting (Vandewater et al., 2005; Lee, 2013). As such, these parental variables may represent promising intervention targets.

There is evidence that home-based interventions can be effective for helping parents modify routines and improve health habits (Haines et al., 2013). Interventions such as Healthy Habits, Happy Homes, which uses motivational interviewing and individually tailored counseling by health educators to encourage behavior change has been used to reduce the risk of obesity in at-risk American families. Incorporating a screen use mediation component to home visits may further help parents manage their child’s screen use and increase health promoting behaviors. In particular, interventions could be designed to help parents implement goals and follow a schedule that restricts child screen use.

Several limitations should be discussed. First, the present findings are based on a low-risk, convenience sample facing low levels of sociodemographic risk. As such, replications with larger, more diverse samples are warranted. Our findings are also potentially limited by shared informant bias since parents provided data on media management strategies and child screen time. In terms of strengths, our study was able to examine prospective associations between parent screen mediation strategies and later child adherence to screen time recommendations, above and beyond pre-existing screen time. As such, this study also helps identify modifiable parent-level protective factors for following screen time recommendations.

In conclusion, our results indicate that parent rule and limit setting, but not active forms of mediation that involve coviewing or discussing screen media with preschools, may help children develop healthy media habits. Furthermore, parental restriction appears to be effective above and beyond child screen time habits and child and parent risk factors. Better understanding how naturally occurring real world parental behaviors contribute to child media habits is helpful for designing effective ecologically valid interventions.

## Data availability statement

The data presented in this article are not readily available. As per the participant consent form, data are only available to the research team. Requests to access the data should be directed to caroline.fitzpatrick@usherbrooke.ca.

## Ethics statement

The studies involving humans were approved by Comité d’étique de l’Université de Sherbrooke. The studies were conducted in accordance with the local legislation and institutional requirements. Written informed consent to participate in this study was obtained from the participants’ legal guardian/next of kin.

## Author contributions

EC: Validation, Writing – review & editing. JB: Methodology, Writing – review & editing. GG-C: Conceptualization, Formal analysis, Writing – review & editing. CF: Conceptualization, Formal analyses, Writing – review & editing, secured funding.
